# A Cross-Modal Perspective on the Relationships between Imagery and Working Memory

**DOI:** 10.3389/fpsyg.2012.00561

**Published:** 2013-01-18

**Authors:** Lora T. Likova

**Affiliations:** ^1^The Smith-Kettlewell Eye Research InstituteSan Francisco, CA, USA

**Keywords:** visual imagery, visuo-spatial sketchpad, working memory, primary visual cortex V1, drawing, fMRI

## Abstract

Mapping the distinctions and interrelationships between imagery and working memory (WM) remains challenging. Although each of these major cognitive constructs is defined and treated in various ways across studies, most accept that both imagery and WM involve a form of internal representation available to our awareness. In WM, there is a further emphasis on goal-oriented, active maintenance, and use of this conscious representation to guide voluntary action. Multicomponent WM models incorporate representational buffers, such as the visuo-spatial sketchpad, plus central executive functions. If there is a visuo-spatial “sketchpad” for WM, does imagery involve the same representational buffer? Alternatively, does WM employ an imagery-specific representational mechanism to occupy our awareness? Or do both constructs utilize a more generic “projection screen” of an amodal nature? To address these issues, in a cross-modal fMRI study, I introduce a novel Drawing-Based Memory Paradigm, and conceptualize drawing as a complex behavior that is readily adaptable from the visual to non-visual modalities (such as the tactile modality), which opens intriguing possibilities for investigating cross-modal learning and plasticity. Blindfolded participants were trained through our Cognitive-Kinesthetic Method (Likova, 2010a, 2012) to draw complex objects guided purely by the memory of felt tactile images. If this WM task had been mediated by transfer of the felt spatial configuration to the visual imagery mechanism, the response-profile in visual cortex would be predicted to have the “top-down” signature of propagation of the imagery signal *downward* through the visual hierarchy. Remarkably, the pattern of cross-modal occipital activation generated by the non-visual memory drawing was essentially the inverse of this typical imagery signature. The sole visual hierarchy activation was isolated to the primary visual area (V1), and accompanied by deactivation of the entire extrastriate cortex, thus ’cutting-off’ any signal propagation from/to V1 through the visual hierarchy. The implications of these findings for the debate on the interrelationships between the core cognitive constructs of WM and imagery and the nature of internal representations are evaluated.

## Introduction

Mapping the distinctions and interrelationships between imagery and working memory (WM) remains challenging. Although each of these major cognitive constructs is defined and treated in various ways across studies, most accept that both imagery and WM involve a type of internal representation available to our awareness; in WM, however, there is a further emphasis on goal-oriented, active maintenance and use of this conscious representation to guide voluntary action.

WM refers to the temporary storage and manipulation of information, and is invoked as the mechanism for information processing during the performance of a wide range of everyday tasks (e.g., Baddeley and Hitch, 1974; Baddeley, 1986, 1992, 2000, 2003; Logie et al., 1989; Logie and Marchetti 1991; Logie 1995; Baddeley and Andrade, 2000). Initially, the proposed structure included the central executive component and two active storage buffers – the *visuo-spatial sketchpad* and the *articulatory/phonological loop*. While the visuo-spatial sketchpad is considered to be responsible for the temporarily storage and manipulation of visuo-spatial material, the phonological loop is posited to provide a similar function for verbal material. An enhanced version of the multicomponent WM model added an *episodic buffer* (Baddeley, 2003).

Interestingly, the involvement of the early visual cortex, and area V1 in particular, has been a critical issue in discussions both on the neural substrate of the putative visuo-spatial sketchpad and on the nature of imagery. The V1-substrate propositions are based mainly on the fact that, although most areas in occipital cortex are topographically organized, area V1 has the unique status of being the *largest topographic map* in the brain, with the *highest spatial resolution*, in addition to parallel processing of the information from the whole map surface (in contrast to the sequential processing in some other modalities).

All of these features are critically important for a successful “sketchpad” implementation, which is why previous theoretical as well as neurophysiological studies in non-human primates (e.g., Mumford, 1991, 1996; Lee et al., 1998; Super et al., 2001a,b; Lee and Mumford, 2003; Super, 2003) had suggested V1 as the source of the high-resolution visuo-spatial “sketchpad” function: “instead of being the first stage in a feedforward pipeline, V1 is better described as the unique high-resolution buffer in the visual system” (Lee and Mumford, 2003).

However, the same characteristics of this region are also key requirements for the existence of a pictorial-code form of visual imagery. Thus, the issue of V1 involvement has been central in the long-standing debate about the nature of visual imagery, which relates to the question of whether the imagery “code” is pictorial or propositional (Kosslyn et al., 2001; Kosslyn and Thompson 2003).

Neuroimaging is a valuable tool that can help to resolve these issues, but while there has been much neuroimaging work on imagery, this is not the case with the putative WM sketchpad. The neural substrate for visual imagery has been found to largely *overlap* with that for visual perception (e.g., Ishai and Sagi, 1995; Kosslyn et al., 1999; Kreiman et al., 2000; O’Craven and Kanwisher, 2000; Kosslyn et al., 2001; Kosslyn and Thompson 2003; Mechelli et al., 2004), with the activation pattern implying that the signal propagates from higher cortical regions in a *top-down* manner through the visual hierarchy toward V1. The resultant top-down gradient of activation provides a notable signature of the visual imagery activation pattern; consistent with this employment of the visual pathway for imagery, there is no significant negative signal (i.e., no flow interruption) for imagery in occipital cortex (Ganis et al., 2004).

While a substantial activation in the higher areas has been consistently found across the imagery studies, this has not been the case with V1. Although some level of V1 activation during imagery has been reported in several studies (e.g., Kosslyn et al., 1993; Le Bihan et al., 1993; Sabbah et al., 1995; Kosslyn et al., 1996; Chen et al., 1998; Shin et al., 1999; Thompson et al., 2001; Ishai et al., 2002; Lambert et al., 2002; Ganis et al., 2004), a larger number of studies did not find any V1 activation at all (e.g., Goldenberg et al., 1991; Charlot et al., 1992; Mellet et al., 1995, 1998a,b; D’Esposito et al., 1997; Ishai et al., 2000; Knauff et al., 2000; Trojano et al., 2000; Wheeler et al., 2000; Formisano et al., 2002; Sack et al., 2002; Mazard et al., 2004; Kaas et al., 2010). Importantly, even when V1 was activated during imagery, the signal there was *significantly weaker* than in the extrastriate visual areas. Thus, the level of V1 activation is of great importance for the imagery debate, as imagery activation in V1 implies the usage of a pictorial code. Kaas et al. (2010), however, whose primary goal was “to eliminate the effects of (short- or long-term) memory in the investigation of the effects of mental imagery,” conclude that their results “suggest that the activation in early visual areas observed in previous imagery studies might be related to short- or long-term memory retrieval of specific sensory experiences.”

To analyze the disparate results on the V1 involvement in the imagery literature, Kosslyn and Thompson (2003) defined sets of variables associated with each of three theories, which were then fit to the observed results using logistic regression analysis to discover how well each theory predicted when early visual cortex was activated. The three theories were Perceptual Anticipatory Theory (pictorial imagery coding), Propositional Theory (non-pictorial propositional imagery coding), and Methodological Factors (determining factors, such as low neuroimaging resolution or differential resting activation in postulated imagery loci, that may need to be controlled in order to resolve imagery-specific activation). Their analysis identified three variables that optimally predicted the differences in the probability of activation across imagery studies. Notably, two of the variables were *task-dependent* requirements (the requirement to note *high-resolution details* in the stimuli and the requirement to visualize *shapes* rather than abstract spatial relations), while the third was purely technical (sufficiently high sensitivity of the technique).

Thus, the operation of the (pictorial) visual imagery and of the visuo-spatial sketchpad concepts share (i) similar task requirements, and (ii) similar need of V1 usage (although note that, in contrast to the putative sketchpad, the top-down theories of imagery are not restricted to V1 but require activation of the whole visual hierarchy). A number of behavioral studies have addressed possible interactions between WM and visual imagery (e.g., Bruyer and Scailquin, 1998; Baddeley, 2000), with the most recent by Keogh and Pearson (2011) suggesting an imagery-dependent dichotomy in cognitive strategies for visual WM.

### Questions

Despite the array of studies on the issue, the above review indicates that the neural substrates as well as the interactions between these two cognitive constructs are still far from being definitively resolved. In particular, the visuo-spatial sketchpad remains an almost entirely theoretical construct. If there is a visuo-spatial “sketchpad” for WM, does imagery use the same representational buffer? Alternatively, does WM employ an imagery-specific representational mechanism to occupy our awareness? Or do both constructs utilize a more generic “projection screen” of an amodal nature?

### A novel approach

#### The drawing-based memory paradigm

Likova (2010a, 2012) recently conceptualized the drawing task as the basis for a novel memory paradigm to address these questions. Drawing, and in particular memory-guided drawing, challenges both the encoding of detailed spatial representations and their explicit retrieval from memory for “projection” back onto a mental high-resolution “screen” to guide the movements of the drawing hand with the requisite precision. A cortical region such as V1 would be an ideal neural implementation of the required “screen”; thus, the putative *V1* *visuo-spatial sketchpad* is a plausible theoretical construct that provides for memory retrieval for just the kinds of spatial representations involved in the drawing task, allowing for the active maintenance of information about stimuli no longer in view. (It is not by chance that this ubiquitous tool of the real drawing process – the use of a disposable sketchpad – was the metaphor employed for the memory module in question.)

Importantly, the drawing-from-tactile-memory task effectively transcends simple “recognition memory.” An easy demonstration makes the point: Close your eyes and try to imagine the objects on your desk, the face of your close friend, or even your own face; in particular, try to “see” the detailed shapes as though you are going to draw them. It is amazing how misleading the feeling is that we “know” any of those very well. Despite the fact that we would effortlessly recognize them (*recognition memory*), when we try to retrieve the details, they fade or somehow escape our grasp, although we do not feel these information gaps when recognizing immediately the object as a whole. Many early representational details seem to be lost along the passage through the visual pathway before they have been integrated into the internal reconstruction of the face/object that they represent. In other words, recognition memory seems to operate at the higher-level of object category processing and does not need to retain the more “local” level of detail. In contrast, a highly detailed kind of spatial memory is engaged to meet the needs of the efferent drawing task; this “*memory-for-drawing*” preserves and recalls details sufficient to enable the complex spatiomotor act of producing an accurate drawing.

#### Conceptual framework

Both the novel Drawing-Based Memory Paradigm and the Cognitive-Kinesthetic Training Method, are based on a framework of principles (Likova, 2012), including:

1. *Space transcends any specific sensory modality*. As emphasized by the phenomenon of drawing by the blind (e.g., Kennedy, 1993, 2000; Kennedy and Igor, 2003; Kennedy and Juricevic, 2006; Ponchillia, 2008; Likova, 2010a, 2012), space, and spatial structure are not represented solely by the visual modality. The visual system is best suited to process spatial information, but it is not the only one. Thus, when deprived of visual input, the brain is capable of employing the “free” visual resources in the most relevant way. (As there is an ambiguity in the use of the term “spatial,” particularly in the WM and imagery literature, note that when used in this paper, “spatial” refers to the perception of any spatial structure – 2D or 3D, static or dynamic – *independently* of the sensory modality exploring it. For example, a face can be recognized by exploring its spatial structure with the hands, or a geometric function can be “seen” by audio-graphics, etc.) My view is that *drawing* deals with spatial structures in this general sense, and consequently it has the advantage that can readily be “translated” from a visual into a tactile form.
2. *Closing the perception-cognition-action loop is a powerful amplifier for learning*, so the *task* selection is critical. *Drawing* is a complex task precisely orchestrating multiple brain mechanisms, and consequently, it provides for an integrative, perception-cognition-action paradigm.
3. *Training in highly engaging* *unfamiliar* *task*s that provide *fun and inspiring outcomes* is a fruitful paradigm for driving brain reorganization and assessing its earliest stages. *Drawing* inherently embodies all of these components, particularly when studied under the circumstances of visual deprivation (which are unusual for a “visual” art); the characteristic for drawing sense of completion, creativity, and fulfillment amplify the experience-based plasticity.
4. *Tasks demanding detailed re-expression of memory-representations force the development of precise and robust memory*. *Drawing*-from-memory demands such explicit *re-expression* through the motor loop, and consequently it demands “high-resolution” internal representations to be communicated back through the drawing act.
5. *Studies on memory would highly benefit from tasks providing “direct” memory-control*. *Drawing*-from-memory incorporates such direct control by providing direct memory “readout,” as it ensures an explicit expression of the remembered information by externalization of the mental representation that guides the drawing hand.

These considerations led me to the choice of *non-visual drawing*, which incorporates all of the above principles, as a paradigm for both *training* and *studying* cross-modal memory. The role of WM and imagery in mediating the training effects were evaluated by functional Magnetic Resonance Imaging (fMRI).

#### The cognitive-kinesthetic drawing method

To employ this novel memory paradigm for studying learning-based plasticity, a method is needed to train non-sighted people to draw not simply without visual feedback, but guided solely by *non-visual* memory. Recently I have developed a novel technique, the Cognitive-Kinesthetic Drawing Method, which proved to be very effective in the successful training of people under total visual deprivation. Congenitally blind, late-onset blind and blindfolded were successfully trained in only a week of 1.5 h/day sessions to draw complex face and object structures (as opposed to simple geometric or grid structures), guided solely by tactile-memory (Likova, 2010a,b, 2012).

In contrast to standard imaging studies of tactile activation, the fMRI evaluation that was run before and after the training was specifically designed to probe the *memory* involvement by recording the brain activity while drawing-from-memory in the absence of any visual or tactile input from the learned raised-line drawing templates (see Materials and Methods). This novel Drawing-Based Memory Paradigm has the unique advantage of providing an *explicit* memory “readout” of the specific memory representation that guides it. Importantly, the Cognitive-Kinesthetic training allows subjects to learn to draw from memory the *specific memorized* objects and faces that they had explored, not just some long-standing “clichés,” thus showing that the particular memory-representations generated during the *tactile exploration* phase were guiding their drawing.

To provide for comparative pre/post-training analyses of brain activation, an innovative platform was developed including the first MRI-compatible multisensory drawing tablet, with a stylus incorporating a fiber-optic motion-capture system to record the drawing movements in the scanner for off-line analyses.

These unique capabilities allow for testing between the following hypotheses:

##### Hypothesis I

If the memory-based drawing task is mediated by a transfer of the *tactually* felt spatial configuration to the *visual imagery* mechanism, the predicted response-profile in the visual cortex would have the top-down “imagery signature” of propagation of the imagery signal *downward* through the visual hierarchy, with activation significantly *decreasing* from the higher extrastriate areas toward V1.

##### Hypothesis II

If V1 was being employed as a WM sketchpad *independently* from the visual imagery process, it would be activated by a *separate pathway* external to the visual hierarchy, together with activation of WM-related sites beyond the occipital lobe. Moreover, if V1 activation is found in *non-sighted* drawing, it will also confirm a re-conceptualization of the sketchpad buffer from being visuo-spatial to being independent from sensory modality, or *amodal* as previously proposed *on the basis of a study in the congenitally blind* (Likova, 2012).

## Materials and Methods

### Experimental design

**Figure 1:**
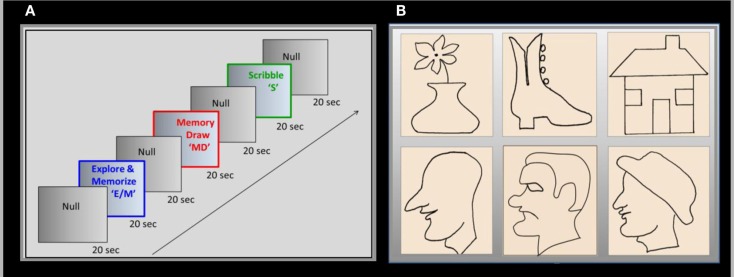
**Experimental design**. **(A)** Drawing was investigated in a three-phase paradigm consisting of a memory-guided drawing task, abbreviated as “MemoryDraw” (*MD*), plus two control tasks: a motor-control and negative memory-control task “Scribble” (*S*), and a task of perceptual exploration and memorization of the model to be drawn “Explore/Memorize” (*E/M*). Each task’s duration was 20 s, with 20 s null intervals interposed between the tasks, the whole 140 s trial sequence being repeated 12 times in each scanning session using a new image for each repeat. **(B)** Raised-line drawings of realistic faces and objects were presented as templates to be explored by the subject using her left hand. The quality of the reproductions was assessed by a masked rating procedure, based on recognition and similarity to the templates (examples of reproduction are shown in Figure 3).

A battery of raised-line models of faces and objects was developed as the drawing targets in a three-task block fMRI paradigm, with interleaved baseline conditions (Figure 1A). The three tasks were as follows: *Explore/Memorize* (*E/M*) – perceptual exploration and memorization of the model to be drawn; *MemoryDraw* (*MD*) – a memory-guided non-visual drawing task; and *Scribble* (*S*) – a motor-control and negative memory-control task. Each task duration was 20 s, with a 20 s baseline condition [*NullInterval* (*NI*)] intervening between the tasks. Importantly, as opposed to the usual null periods, the subjects not only rested motionless but were instructed and practiced to clear any memory or image structure from awareness (“mind-blank”). The start of each task or null interval was prompted by an auditory cue. The whole three-task sequence with interleaved null intervals (*NI*, *E/M*, *NI*, *MD*, *NI*, *S*, *NI*) was repeated 12 times in each 1-hour fMRI session using a new image for each repeat.

In *Explore/Memorize*, using the left hand only, the subjects had to tactually explore a raised-line drawing model on the left slot of the drawing tablet, and to develop a full memory representation of the image in preparation for the *MD* task. Then the model image was removed, and the subjects rested motionless for 20 s with no image in mind (*NI*). In the following *MD* phase the fiber-optic stylus was used to draw the image (from tactile-memory) on the right slot of the tablet using only the right hand; the trajectory of the stylus tip was recorded with high precision. *Scribble* was a control for both movement-specific activation (due to hand or eye movements) and absence of a memory involvement; the subjects had to move the stylus with the right hand in a random trajectory over the right slot of the tablet matching the extent and rate of the drawing movements, but under instructions to avoid planning or imagining any particular trajectory or cognitive content.

#### Rational of the drawing requirements

(i) The requirement that the models were always explored with the left hand but drawn by the right hand was an advanced aspect of the experimental design ensuring that in the *MD* task the cortex controlling the right (drawing) hand did not have any direct “haptic knowledge” of the image. This design enforces the development of a *detailed memory* representation in order to transfer the information later to the opposite (drawing) hand. (ii) Furthermore, the left hand was not allowed to follow the contour being drawn by the right hand, ensuring that the subjects learned to draw from memory *without* relying on specific tactile feedback of the raised-line configuration. Moreover, the reliance on detailed enough memory was further guaranteed by the fact that the virtual stylus left no tactually perceivable trace, eliminating any possibility for tactile tracing during drawing in the scanner. Together, these design features enforce the encoding of a robust *spatial* *memory* representation needed to guide the drawing trajectory. The quality of the reproductions was assessed by a masked rating procedure, based on recognition and similarity to the templates.

### Tactile stimulus presentation and hand movement control

#### Multisensory MRI-compatible drawing system

**Figure 2:**
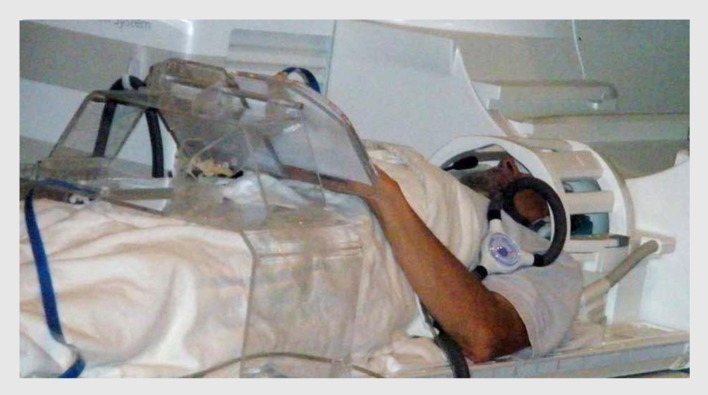
**A subject on the scanner bed operating our novel multimodal MRI-compatible drawing system**. The plexiglass gantry supports a drawing tablet while a fiber-optic drawing stylus captures and records the drawing movements with high precision. The motion-capture information synchronized with the fMRI allows the effect of behavioral events to be analyzed to high precision.

To run drawing studies in the scanner is not a conventional protocol and faced a lot of challenging technological problems. We developed a multisensory drawing-system that: (1) is MRI-compatible, (2) is ergonomically adaptable to the small space available inside the scanner bore, (3) allows multiple tactile images to be presented sequentially in the scanner without the need of any operator assistance, (4) captures and records the drawing trajectory with high precision, and (5) provides a real-time visual feedback when appropriate (Likova, 2010a). It incorporates a dual-slot drawing tablet that is height/distance adjustable for the subject’s arm length and a specially adapted version of a fiber-optic device for motion capture of the drawing movements with high-resolution (Figure 2).This unique drawing-system supports the fMRI investigation of both *tactilely* and *visually* guided drawing. It also allows us to record relevant behavioral and feedback events and to correlate them to the brain activation for full off-line analysis.

#### Auditory cue presentation

The auditory stimuli were presented through Resonance Technologies Serene Sound earphones (Resonance Technologies, Salem, MA, USA). To reduce scanner noise, this equipment employs external ear protectors with perforated earplugs that conduct the auditory cues directly into the auditory passage while blocking much of the scanner noise.

### MRI data collection, analysis, and visualization

#### Subjects

The study was conducted on a group of six subjects with normal vision who were blindfolded during the experiments. The subjects ranged in age from 25 to 59 and were four females and two males. All subjects gave informed consent for the experimental protocol approved by the local research ethics committee, Institutional Review Board.

#### FMRI acquisition

MR data were collected on a Siemens Trio 3T scanner equipped with eight-channel EXCITE capability, a visual stimulus presentation system, response buttons. A high-resolution anatomical (*T*1-weighted) volume scan of the entire brain was obtained for each subject (voxel size = 0.8 mm × 0.8 mm × 0.8 mm). The fMRI blood-oxygenation-level-dependent (BOLD) responses were collected with EPI acquisition from the whole head coil. There were 34 axial slices at 2 s TR, with TE of 28 ms and flip angle of 80°, providing 3.0 mm × 3.0 mm × 3.5 mm voxels throughout the brain. The functional activations were processed for slice-time correction and motion correction. An anatomical segmentation algorithm (mrGray) was applied to the T1 scan, ensuring localization of the signal within the cortical gray matter close to the activated neurons and greatly reducing the blood drain artifacts of BOLD signals displaced from the neural activation sites, which afflict studies in which cortical segmentation is not used.

#### FMRI time-course analyses

The analysis software was Stanford Vision and Imaging Science and Technology (VISTA) software. The data were analyzed to estimate the effective neural activation amplitudes (for each task across the 12 repeats of the three-task sequence in the 1-h scan) by the following procedure. A General Linear Model (GLM) consisting of a (3 + 1)-parameter boxcar neural activation model convolved with an estimated hemodynamic response function (HRF) was fitted to the BOLD responses (i.e., a 1-parameter boxcar for each of the three tasks, plus a 1-parameter 8-boxcar sequence to model the auditory cue presentations). An additive fourth-order polynomial was applied to capture low-frequency drift in the BOLD signal. (The HRF parameters were determined once per session by optimizing this model to a subset of gray matter voxels identified as most responsive to the task/null alternation frequency in this experiment.) Thus, the parameters of the activation model consisted of the boxcar activation amplitudes for the three-task periods, relative to the remainder of the 140 s scan duration.

#### Voxel-wise parametric maps

For each task statistical parametric maps were generated, based on the estimated activation amplitudes from the above GLM in each voxel. As is standard for GLM, the boxcar neural activation model for each 20 s task period was contrasted with the entire remainder of the 140 s scan duration. All three task-models were optimized jointly to the detrended BOLD waveform. Also, for the pre-post comparison, voxel-wise maps of the change in activation following the training were generated, scaled in terms of *z-score* of the pre-post difference signals. These maps could be viewed in the 3D volume or projected onto 3D views of the inflated cortex or flat-maps of cortical regions of particular interest.

#### ROI activation analysis

The effective neural activation amplitudes (bar-graphs) for each condition in each region of interest (ROI) were estimated by the same GLM procedure but now applied to the *average* signal across all voxels within the ROI. This procedure also provided high-quality time courses for evaluation of the response dynamics and its comparison across tasks and stages of training.

The confidence intervals in the bar-graphs were defined by the amplitude variability of the 12 repeats of the three-task sequence in each 1-h scan. The *dashed lines* and the *error bars* represent confidence intervals for *two different forms* of statistical comparison of the activation levels (i.e., of the beta weights for the event types in the GLM): (1) The *dashed lines* represent the 99% “zero” *confidence interval* (*p* < 0.01, uncorrected) within which the activation amplitudes are not significantly different from zero (i.e., relative to the noise variance for no stimulus-related activation defined as the residual variance after the GLM model fit of the *FMRI time-course analyses* section described above); thus this statistical criterion is designed to indicate the significance of each individual activation (at *p* < 0.05, corrected for multiple applications within each figure); (2) The *error bars* are “difference” *confidence intervals* designed to illustrate the *t-test* for the significance of differences *between* activation levels in each figure (i.e., the differences are not significant unless they exceed the confidence intervals for both compared activations). In the text, all ROI-comparisons are specified as significant by the *t-test* using a statistical criterion threshold of *p* < 0.05 corrected for multiple comparisons.

#### Topographic mapping

The boundaries of the retinotopic projection areas V1, V2d, V2v, V3d, and V3v were established as described in Sereno et al. (1995); Tootell et al. (1996); Engel et al. (1997). Retinotopic projection areas V3A, V3B, hV4, and V7 were specified in accordance with Tyler et al. (2005). The retinotopic mapping was done by using standard retinotopic stimuli – expanding rings and traveling wedges. An innovative 14-step procedure (Likova, 2010a) allowed us to warp the brains to the same MNI brain coordinates, within which other localizers could also be specified on the basis of prior studies.

## Results

**Figure 3:**
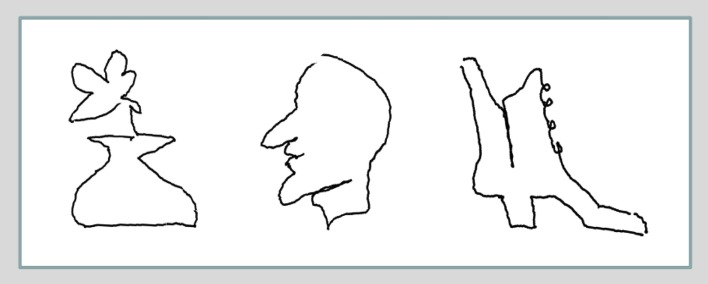
**Examples of blindfolded drawings of the vase with a flower, the face profile, and the boot (the corresponding templates shown in Figure 1B are easy to recognize: the first and the second in the top row, and the first in the bottom row)**. Remarkably, the post-training drawings, recorded in the scanner by the motion-capture system show a lot of specific detail, which makes them readily recognizable as specific examples of their category, although they were drawn without visual or tactile input (i.e., with eye-hand coordination eliminated), but were guided solely by tactile-memory.

The subjects were all able to improve their drawing skills so as to complete each drawing with its particular characteristics within the 20 s allotted for the drawing tasks (which took many times longer before training). Examples of drawing recorded in the post-training scanning session are shown in Figure 3, illustrating the level of detail required to complete each drawing.

### BOLD responses to drawing guided by tactile-memory

**Figure 4:**
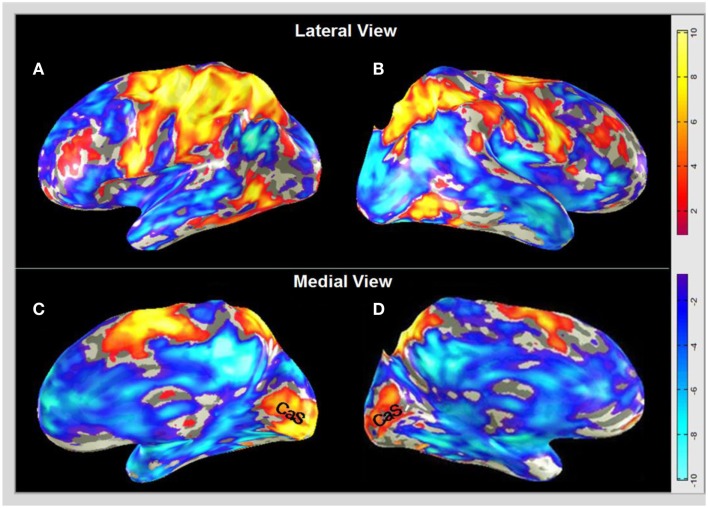
**BOLD activation and deactivation in *non-visual memory drawing* in the blindfolded**. Post-training group responses from the *MD* task are derived according to the GLM described in Section “Materials and Methods,” and projected on inflated representations of the lateral left **(A)** and right **(B)**, and medial left **(C)** and right **(D)** hemispheres in MNI brain coordinates. Dark-gray, sulci; light gray, gyri. **(A,B)** A non-occipital network of temporal, parietal, and frontal regions is activated (yellow-orange coloration), together with strong deactivation (blue-cyan coloration) in a network that corresponds broadly to the default-mode network (except for the occipital lobe portion). **(C,D)** Both medial views show *massive activation* along the *calcarine sulcus* (CaS) corresponding to V1 surrounded by deactivation, which extends throughout the lateral regions of the visual cortex. Activation is shown down to −1 < *z* < 1; the scale bar indicates the color-coding for the respective *z-score* levels. Note that, interestingly, the medial CaS activation spreads to the same eccentricity in both hemispheres.

The initial analysis gives an overview of the averaged cortical activation for the *MD* task in the brains of the group of blindfolded subjects. Figure 4 shows the post-training map, projected on inflated representations of the lateral and medial views of the two hemispheres. As expected, there is strong activation in the pre-motor, motor, and somatosensory cortex in the dorsal regions of the brain (the pre-central and post-central sulci, PreCS and PostCS) predominantly for the left hemisphere controlling the right hand that was performing the drawing task (Figures 4A,B). The anterior and posterior dorsal regions also showed enhanced activation bilaterally, implying enhanced kinesthetic processing for the drawing movements. The supplementary motor area (SMA, on the medial surface), which plays a role in the planning of complex coordinated movements is also activated bilaterally, although again the left hemisphere responds more strongly. The dorsolateral-prefrontal cortex, known to be of key importance in WM, decision-making, executive control, etc. is activated in both hemispheres. Temporal lobe activation can be also seen in the LOtv region suggested to be involved in tactile object processing. The involvement of many of these regions would be very much predicted on the basis of prior studies. There is also an extensive network of deactivation that, *beyond* the occipital lobe regions, largely overlaps with the default-mode network (e.g., Raichle and Snyder, 2007).

What is surprising within the traditional view of brain architecture, however, is the massive activation in the occipital region along the calcarine sulcus (CaS) for this non-visually and not even sensory (neither visually nor tactually) guided task (in the sense that, as described in Methods, the task has been accomplished with no visual input at any stage, and the drawing phase has been accomplished with guidance only by tactile-memory, i.e., with no concurrent sensory input of any form about the image to be drawn). This region corresponds to the location of area V1 and is the key focus of the present analysis. The strong V1 activation (orange-yellow coloration) can be seen on the medial surface in both hemispheres (Figures 4C,D).

Activation is shown down to −1 < *z* < 1 because the lower amplitudes appear to form a consistent fringe around the cluster regions of high significance. Indeed, such a visualization approach is not often taken; however, in cases where not only the positive but the negative clusters are also of interest, this visualization approach allows the following to be shown: (i) whether the surrounding regions similarly show activation but just below the threshold level, or (ii) whether there is an extended cluster of deactivation. Higher-threshold presentations mask such differences; thus, it seems worthwhile to provide maximum information in the figures. For restricting to the individual voxel (rather than cluster) significances, the color-coding provides explicit information as to the activation pattern for any preferred threshold level.

**Figure 5:**
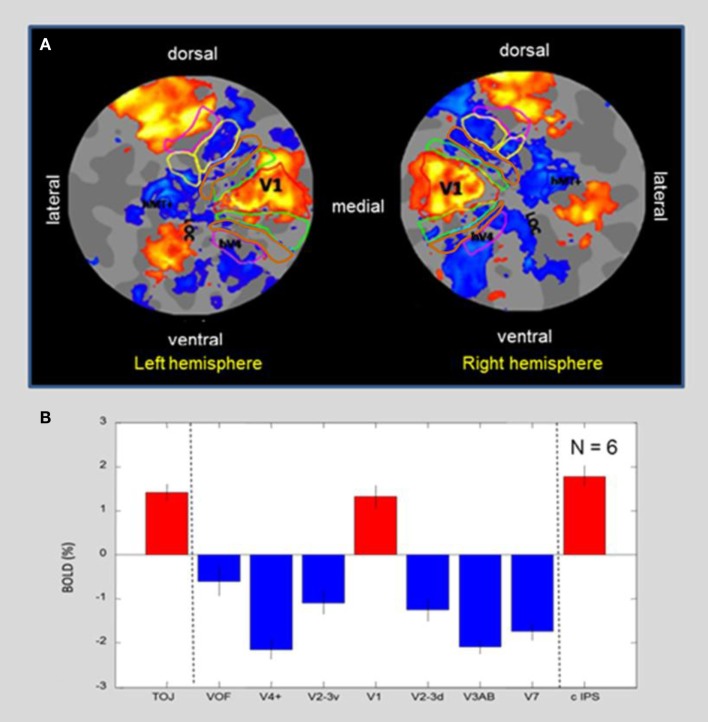
**(A)** *MD* flat-maps centered on the occipital pole. ROIs for the retinotopic hierarchy are indicated by colored outlines, with hMT+ and LOC based on functional localizers. The post-training *MD* map shows a “triad” of three activation regions (orange-yellow coloration). Note in particular that the (non-stimulated visually) primary visual cortex, V1, forms an unusual isolated “island” of *activation* surrounded by a “sea” of *suppression* in the adjacent retinotopic areas. The other two activated regions seen on the flat map are the caudal intraparietal sulcus (cIPS) dorsally and an additional locus at the occipitotemporal border (LOtv). **(B)** Average response amplitude with standard errors for blindfolded *memory-guided drawing* in a group of six subjects, showing positive signal in the triad of areas – primary visual area V1, cIPS, and LOtv; these three “islands” of positive activation are separated by strong deactivation in both the ventral and the dorsal extrastriate areas. Error bars represent 1 standard error of the means.

To better evaluate the *activation/deactivation* pattern in the context of the functional “geography” of the visual cortex, Figure 5A shows a flat-map representation of the occipital cortex of a representative blindfolded subject who had all of the visual-hierarchy areas mapped, as well as the motion complex hMT+ and LOC. The flat-maps are centered on the occipital pole and oriented as if viewed from the back of the head, with activation/deactivation designated as in Figure 4. The retinotopic boundaries were determined in a separate scan using a 20° circular field. Note that the peripheral boundaries of these regions thus correspond to about 10° of eccentricity, which corresponds approximately to the maximum extent of the BOLD activation (yellow-orange coloration). Area V1 is outlined by a red contour. It is remarkable to see that the *tactile-memory* drawing not only generates activation specific to V1 (although no sensory visual or even tactile information about the drawing templates was available), while the entire extrastriate hierarchy that surrounds V1 is massively *deactivated*. The extended non-visual response in what traditionally is considered as primary “*visual*” cortex was accompanied by activation in a region of cortex at the occipitotemporal border known to be involved in tactile object recognition, LOtv (e.g., Amedi et al., 2001, 2002; Reed et al., 2004); activation is also seen dorsally in the caudal intraparietal sulcus (cIPS).

Average amplitudes across the ROIs shown in Figure 5A are quantified for a group of six normally sighted subjects performing the drawing task under blindfolded conditions in Figure 5B. As in the example subject, the only three regions in this part of the cortex showing significant activation are V1, and the two regions beyond the visual retinotopic hierarchy, LOtv, and cIPS. All the other regions show significant reductions in the BOLD below baseline.

**Figure 6:**
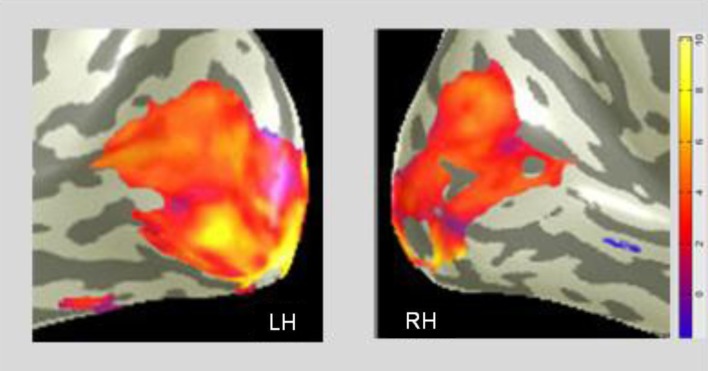
**The *MD* task shows the predominant training effect in the primary visual cortex**. A voxel-wise comparison, projected on inflated representations of the posterior left (LH) and right (RH) hemispheres, with orange-yellow coloration showing the average *pre/post increase* in BOLD activation for *MD* in the CaS (V1) region.

A difference *MD* map, which represents voxel-wise comparison of the *post-training* BOLD activation relative to the *pre-training* level is presented in the CaS region in Figure 6. It shows a pronounced bilateral increase of activation after the Cognitive-Kinesthetic training.

### Cross-task comparison of V1 activation

**Figure 7:**
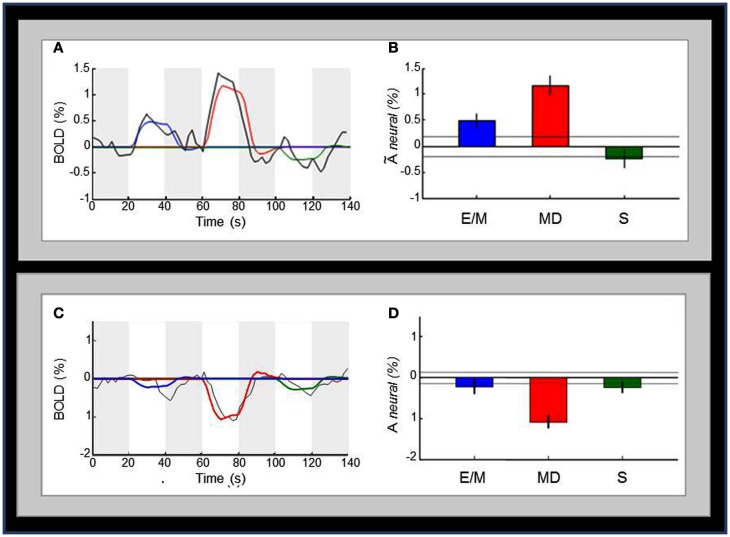
**Comparison of the activation pattern across the three tasks**. **(A)** Average time-courses of BOLD activity (black curve) in V1 for the sequence of the *E/M*, *MD*, and *S* task intervals (white bars); the colored curves are the best fits of the model predictions for the three tasks to the time course; the four dark-gray bars indicate the 20 s null intervals separating the three tasks. **(B)** Bar-graphs for the estimated V1 activation for each task; the activation levels refer to the beta weights for the event types in the GLM. Dotted lines and the error bars represent confidence intervals for *two different forms* of statistical comparison of the activation levels. *Dotted lines* represent the 99% “zero” *confidence interval*, within which the activations are not significantly different from zero. *Error bars* are 99% “difference” *confidence intervals* designed to illustrate the *t-test* to assess the significance of the differences *between pairs of* activation levels in each figure, i.e., amplitude differences are not significant unless they exceed the confidence intervals for both compared activations. **(C)** Average time-courses of BOLD activity in the deactivated regions surrounding V1. **(D)** Bar-graphs of estimated activation for each task for the deactivated regions presented in **(C)**. Conventions in **(C,D)** are as in **(A,B)**, respectively.

Figure 7A below shows the average time-courses of BOLD activity (black lines) in V1 for the sequence of the three-task intervals (white bars); the four dark-gray bars indicate the 20 s null intervals separating *E/M*, *MD*, and *S* tasks. The bar-graphs in Figure 7B show the estimated activation in each hemisphere for each task. Importantly, the *MD* task (red color), which requires retrieval of *detailed tactile-memory* representation is the one that produces the strongest activation in V1; note that eliminating the memory component (in the “non-memory” drawing task *S*, green color) correspondingly eliminates any response in this purely motor form of the task. Although the *E/M* task (blue color), which represents the memory encoding phase, also seems to employ V1 to some degree, significantly less activation is observed than for the *MD* task. Correspondingly, Figures 7C,D show the average time-courses and bar-graphs for the deactivated extrastriate regions. The *MD* response for these regions is the most prominent and highly significantly negative. *E/M* and *S*, on the other hand, have marginally significant responses, but are not significantly different from each other. All cross-task ROI-comparisons in the text are specified as significant by the *t-test* at a statistical criterion threshold of *p* < 0.05, corrected for multiple comparisons.

Although the time-course plots show that there was no strong activation during the “mind-blank” null periods between the task epochs, to formally assess the degree of memory involvement in V1 following the *E/M* phase, we ran regressors for all null periods, and compared the one after *E/M* with the average for the remainder of the null periods. As expected based on the “mind-blank” design of these periods, there were no significant differences in the V1 ROI (*p* ≫ 0.1), implying that there was no evidence for memory engagement of V1 during this null period. The corresponding analysis was also run on the deactivated extrastriate ROI with the same result.

## Discussion

Employing a novel memory paradigm based on drawing in normally sighted subjects while blindfolded revealed that the primary “visual” area V1 can be strongly activated in a *non-visual WM* task: the task of blindfolded drawing guided solely by memory acquired during haptic exploration of complex spatial structures, such as raised-line objects and faces. Furthermore, the pattern of response showed a number of unique characteristics:

(i) The occipital activation was largely restricted to area V1.
(ii) The strong V1 activation was remarkably well structured, ceasing rapidly at a specific eccentricity.
(iii) Surprisingly, the V1 activation was surrounded by massive *deactivation* of the entire extrastriate visual hierarchy.

In contrast to the parallel nature of typical visual processing, haptic exploration operates in a sequential manner. As the blindfolded are subjects who have an intact visual system, and presumably a well developed visual imagery mechanism, one possibility it is that once the spatiotemporal integration of the sequentially explored template-objects was completed, the memory retrieval was implemented by the visual imagery mechanism (*Hypothesis I*). That is, in principle, it is possible that the memory of the complex spatial structures could in some way be transferred to higher-order visual imagery “processors,” which in turn may have mediated the corresponding conscious experience via a top-down propagation through the visual hierarchy to a high-resolution memory representation in V1.

However, this hypothesis seems not to be supported by the data because, although we find strong activation in the iconic visual area V1, it is implausible that the signal is “delivered” through the visual hierarchy, as this hierarchy is not only not activated but is massively *deactivated*. This implies that V1 is “cut-off” from the higher-level cortical regions that could generate and propagate the imagery signals. It is important to emphasize that this *pattern of V1-activation/extrastriate-deactivation* is quite distinct, almost the *inverse* of the known hierarchical pattern for visual imagery (which is a top-down process strongest in the higher extrastriate areas and *decreasing* toward the lower areas, often not reaching V1 at all; see Introduction). The implications of the deactivation cutting-off V1 from receiving signal through the extrastriate visual pathway go further, beyond simple judgment of “similarity” or “difference” of activation patterns, to imply functional incompatibility with the main principle of visual imagery as a process propagating through the visual pathway downward to V1.

Consequently, the unique pattern of response in the blindfolded is not compatible with an explicit role for visual imagery in this form of WM. Instead, the strong but isolated V1 activation seems to be more consistent with *Hypothesis II* that V1 is operating as a WM component, such as the spatial memory-buffer/sketchpad of the composite WM model.

The *training* paradigm of the current study also provides a *causal* manipulation that links the memory enhancement to the *increase* in V1 activation as a result of the (non-visual) Cognitive-Kinesthetic training, consistent with the memory-buffer interpretation of the role of V1. Moreover an important twist for this interpretation is the lack of any visual stimulation under the blindfolded conditions, implying that activation of the V1 buffer should be independent of the input modality. In other words, the present results imply that the nature of the V1-buffer is not “visuo-spatial” but “*amodal*-spatial”.

**Figure 8:**
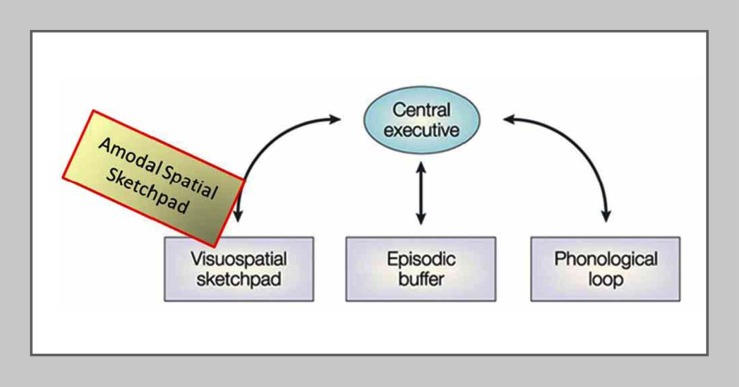
**The proposed re-conceptualization of the *visuo-spatial* sketchpad as an *amodal-spatial* sketchpad**. Modified schematic of the main modules of Baddeley’s classic model of working memory including the visuo-spatial sketchpad (after Baddeley, 2003), where the added “Amodal-Spatial Sketchpad” block depicts our re-conceptualization of the *visuo-spatial* sketchpad as being accessible to any sensory modality (from Likova, 2012).

Importantly, the results in the blindfolded reinforce the previous implication from a parallel study of memory drawing in a congenitally blind subject (Likova, 2012) that V1 was operating as an *amodal* memory-buffer because the subject had had a complete absence of visual experience and visual memory throughout life and had performed the task entirely based on the memory from the tactile input. The strong post-training activation of V1 in that study thus could not meaningfully be attributed to visual imagery, but is more consistent with the hypothesis that V1 uses an amodal-spatial representation in its operation as the putative memory-buffer.Our re-conceptualization of the *visuo-spatial* sketchpad as being *amodal-spatial* is depicted by the yellowish block in Figure 8.

It is important to note, however, that such an interpretation does not mean that V1 is employed for *storage* of the memory trace. Moreover, in contrast to the usual format of a *baseline* condition, we instructed and practiced the subjects to *eliminate any rehearsal* of either the just-explored templates or of any other memory images for the full 20 s duration of each *null* interval. This duration is also too long to account for the known retention time of any iconic image of a memory trace, that is of the order of a second or less (Sperling, 1963; Di Lollo, 1980). Since the drawings were not experienced as spatial images during the *null* interval, they were evidently held in some other, non-conscious storage location until it was needed for the subsequent drawing task.

Further support for the general idea of early sensory areas in human being involved in some form in WM comes from the seminal study of Harrison and Tong (2009), which “demonstrated that early visual areas can *retain* specific information about visual features held in WM.” However, similarly to visual imagery (and *in contrast* to our data), the *whole sequence* of early visual areas – V1, V2, V3, V3A–V4 was activated in that study. Thus, as should be expected for a visual process, *visual* WM did *not* suppress but activated the extrastriate areas, i.e., it did *not* “cut-off” the V1 signal propagation through the visual pathways. Their result of “*widespread* activity throughout the early visual system” makes it clear that *visually* driven WM uses pathways different from those in our *non-visually* driven WM task, which had *deactivated* all of the extrastriate visual areas.

Thus, visual WM can not account for our data, while on the other hand, our concept of an amodal (modality-independent) spatial-sketchpad in V1 is consistent with the V1 involvement in both visual and non-visual WM, as its amodal nature implies availability through the visual as through the tactile modality. Therefore, the present results encourage an *expanded* view of the V1 functionality to a *cross-modal involvement in WM processing* (the access to which, however, would require different pathways for different modalities).

### Generalization beyond the sketchpad: V1 as a generic “projection screen” of an amodal nature?

Although the present results are consistent with our Hypothesis II, namely that V1 was operating as the neural substrate for the putative WM sketchpad in the MD task, and not as a visual imagery component, they have further significance beyond this specific dichotomy.

In a more general sense, the current results provide a strong demonstration of massive employment of V1 in a higher-order cognitive task that involves *no* visual (or even tactile) *sensory* stimulation. Thus, in principle, these results do not exclude a *broader hypothesis*, specifically that V1 may play the role of a more *generic* “*projection screen*” of an *amodal* nature, which could be utilized by each of the two main cognitive constructs discussed here, as well as by visual WM and by other forms of cognitive functions requiring such a high-resolution “projection screen.” Depending on the specific *task needs*, it may be utilized in either a cross-modal or intramodal manner.

### Possible mechanisms

The general field of visuotactile interactions, especially with respect to primary visual cortex in non-visual sensory stimulation, began from studies in the blind (e.g., Sadato et al., 1996; Cohen et al., 1997; Zangaladze et al., 1999; Pascual-Leone and Hamilton, 2001; Block, 2003). Although further analyses are needed to investigate what underlying mechanisms may mediate the cross-modal activation observed in V1 in the current tactile-memory task, there is a range of general theoretical possibilities, such as unmasking of pre-existing inter-region connections, changes in synaptic weights, modulation of long-range intercortical influences, up-regulation of non-local transmitter sources, or a variety of subcortical mechanisms (e.g., Florence and Kaas, 1995; Jones, 2000; Pascual-Leone and Hamilton, 2001; Raineteau and Schwab, 2001; Block, 2003; Merabet et al., 2008; Van Brussel et al., 2011).

Indeed, the extent of V1 connectivity is currently undergoing an extensive re-evaluation. Recent electrophysiological and anatomical studies in non-human primates reveal a picture of multiple reciprocal connections at lower hierarchical levels, including the primary areas. In addition to the well-known direct feedback projections to V1 originating from the visual hierarchy (Perkel et al., 1986; Ungerleider and Desimone, 1986a,b; Shipp and Zeki, 1989; Rockland, 1994; Budd, 1998; Barone et al., 2000; Suzuki et al., 2000), there are direct feedforward projections to V1 originating from a variety of subcortical structures, including the pulvinar, LGNd, claustrum, nucleus paracentral, raphe system, locus coeruleus, and the nucleus basalis of Meynert (Ogren and Hendrickson, 1976; Rezak and Benevento, 1979; Graham, 1982; Blasdel and Lund, 1983; Doty, 1983; Perkel et al., 1986; Lachica and Casagrande, 1992; Hendry and Yoshioka, 1994; Adams et al., 2000; Schmolesky, 2000). In principle, any of these subcortical structures could be involved in the processes of memory storage and retrieval for the performance of the high-resolution drawing task.

Additionally, Clavagnier et al. (2004), examined feedback projections to area V1 using retrograde tracer injections. Notably, in addition to well-known areas and a number of long-distance feedback connections originating from auditory (A1) and multisensory (STP) cortices, they also found connections from a perirhinal area. The perirhinal-to-V1 connections appear of particular interest (Likova, 2012) in the context of our finding of a memory-related role for V1, as the perirhinal area is adjacent to the hippocampus and has a well-established role in memory storage and retrieval.

These connections therefore could represent another potential pathway for the involvement of V1 in WM and the active processing of stored spatial information. Nevertheless, none of the multiple connections above directly predicts the way that V1 is *cut-off* from the surrounding visual hierarchy by the deactivation. Definitive studies on these issues remain to be conducted.

## Conclusion

This novel experimental approach, showing how a WM task accesses the highest resolution topographic map in the brain (V1) even under non-visual conditions, provides a “real-life” yet tractable paradigm for addressing the role of such high-order cognitive processes in a cross-modal manner. V1 was activated in an isolated fashion in the drawing-from-memory task, supporting our Hypothesis II that V1 operates as the active spatial “sketchpad” underlying the accurate drawing performance under non-visual conditions. The blindfolded drawing results were also consistent with the previous conclusion from a congenitally blind study (Likova, 2012) that the spatial WM sketchpad may operate in an *amodal* (rather than exclusively visual) fashion. The converse hypothesis that the memory retrieval would activate the visual hierarchy in a top-down fashion (as would be expected if this task were mediated through the imagery network), was not supported by the data. In a more general sense, the results are a strong demonstration of a massive *cross-modal* *activation in V1* in a high-level cognitive function. In combination with our previous work with the Drawing-Based Memory Paradigm and training effects of the Cognitive-Kinesthetic protocol, these studies further propel the emerging re-conceptualization of brain architecture as highly interactive and capable of plastic reorganization even after short-term sensory deprivation.

## Conflict of Interest Statement

The author declares that the research was conducted in the absence of any commercial or financial relationships that could be construed as a potential conflict of interest.
